# Acute myeloid leukemia in *SRP54*‐mutated congenital neutropenia

**DOI:** 10.1002/jha2.413

**Published:** 2022-03-16

**Authors:** Anthony Sabulski, David D. Grier, Kasiani C. Myers, Stella M. Davies, Jeremy D. Rubinstein

**Affiliations:** ^1^ Division of Bone Marrow Transplantation and Immune Deficiency Cancer and Blood Diseases Institute Cincinnati Children's Hospital Medical Centre Cincinnati Ohio USA; ^2^ Department of Paediatrics University of Cincinnati College of Medicine Cincinnati Ohio USA; ^3^ Division of Pathology Cincinnati Children's Hospital Medical Centre Cincinnati Ohio USA; ^4^ Division of Oncology Cancer and Blood Diseases Institute Cincinnati Children's Hospital Medical Centre Cincinnati Ohio USA

**Keywords:** congenital neutropenia, pediatric leukemia, hematopoietic cell transplant

## Abstract

*SRP54* mutations have recently been implicated in congenital neutropenia (CN) and the in‐frame deletion, p.Thr117del, is the most common pathogenic mutation reported. The largest study of *SRP54*‐mutated CN to‐date followed 23 patients for a median of 15 years. No patients developed a hematologic malignancy in that study. Given the known risk of leukemia in other CNs it is crucial to know whether patients with *SRP54*‐mutated CN have an increased risk of leukemia. We report the first case of leukemia in a patient with *SRP54*‐mutated CN. A 15‐year‐old male with *SRP54*‐mutated CN (p.Thr117del) was diagnosed with acute myeloid leukemia with myelodysplasia‐related changes on a screening bone marrow evaluation. Next generation sequencing of the leukemia cells identified *CSF3R* and *RUNX1* mutations. These mutations commonly co‐exist in CN‐associated malignancies and suggest leukemogenesis in *SRP54*‐mutated CN may occur in a similar manner to other CNs. He was successfully treated with CPX‐351 followed by hematopoietic cell transplant (HCT) and remains in remission at a follow‐up time of 9 months. Although conclusions from this single report must be limited, this has potentially significant implications for both screening and treatment practices for these patients, including the role and timing of HCT.

## 1 INTRODUCTION

The *SRP54* gene encodes signal recognition particle (SRP) 54 GTPase protein and mutations in *SRP54* have been linked to congenital neutropenia (CN) and a Schwachman‐Diamond‐like syndrome [1]. Bellanne ´‐Chantelot et al. [1] described 23 subjects with *SRP54* mutations who had neutropenia as well as neurodevelopmental delay and exocrine pancreatic deficiency. *SRP54* is one of at least 25 known genes implicated in CN, many of which have an increased risk of hematologic malignancies [2]. *SRP54‐*mutated CN can result in lethal infections and patients rely on exogenous granulocyte colony stimulating factor (G‐CSF) to limit infections. At least six patients with *SRP54* mutations have undergone hematopoietic cell transplant (HCT) [1, 3, 4, 5]. None of these patients was transplanted for a hematologic malignancy and there have been no published reports of hematologic malignancies in patients with *SRP54* mutations. We describe our experience with the first reported case of leukemia in a patient with *SRP54‐*mutated CN.

## 2 METHODS

All clinical data were compiled prospectively.

## 3 RESULTS/DISCUSSION

**FIGURE 1:**
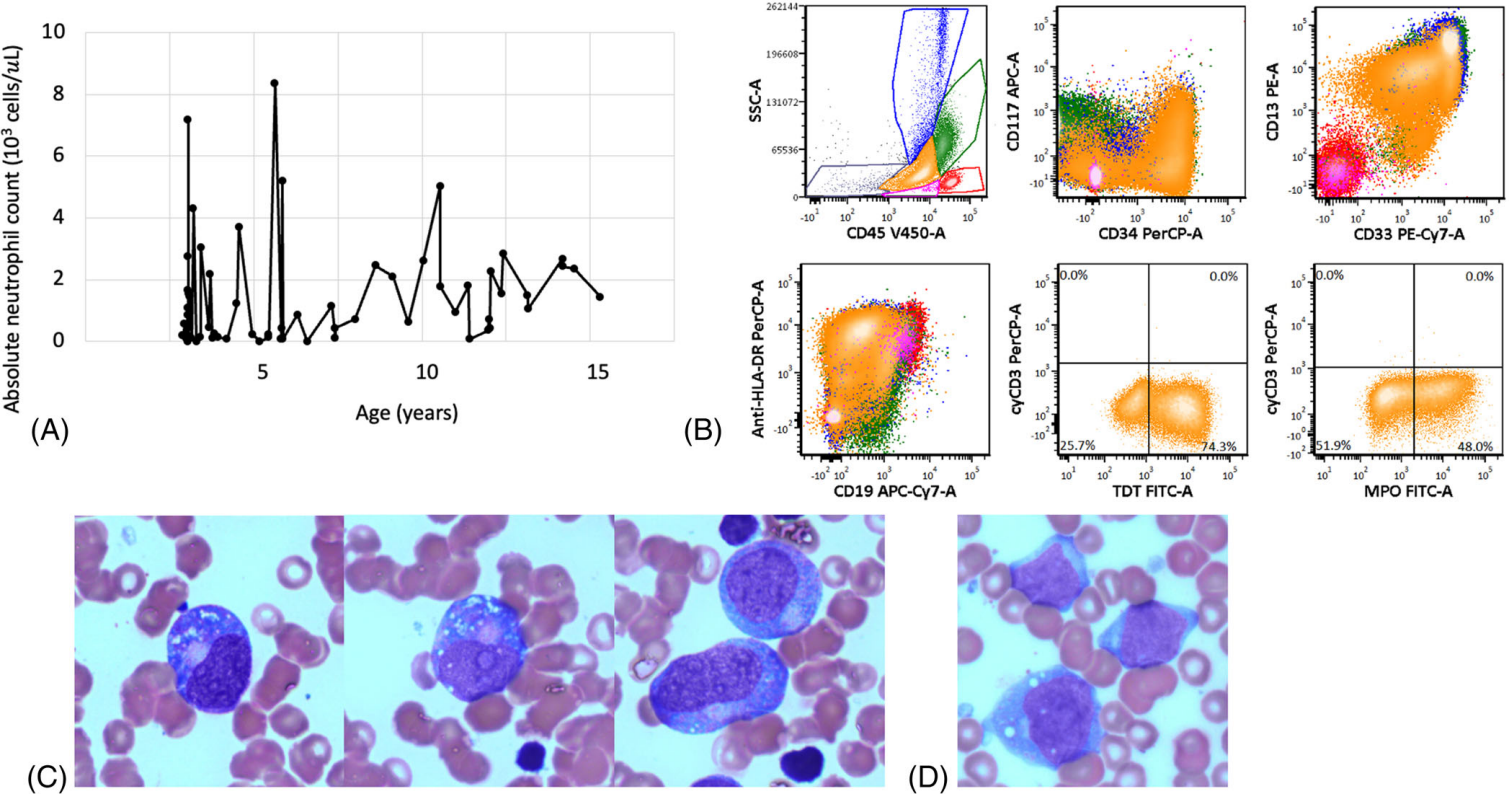
Neutrophil trend and diagnostic findings in a patient with SRP54‐mutated CN and acute myeloid leukemia (AML). (A) Absolute neutrophil count (ANC) trend starting at 2 years of age and up until 15 years of age when the patient was diagnosed with leukemia. (B) Flow cytometry reveals a population of myeloid blasts that express CD34, variable CD117, CD13, CD33, HLA‐DR, partial TdT, and partial myeloperoxidase. (C) Myeloid precursors from a surveillance marrow showing cytoplasmic granules and condensed granulation located in the Golgi. (D) Blasts at the time of leukemic transformation

A 15‐year‐old male with CN secondary to a heterozygous *SRP54* mutation (p.Thr117del) underwent an annual surveillance bone marrow aspirate and was found to have 38% myeloblasts (Figure 1). The patient had no prior history of myelodysplasia or blast populations on surveillance bone marrow studies. He was diagnosed with CN at 2 months of age when he was found to have an absolute neutrophil count (ANC) of 0 × 10^3^ cells/μl and a known maternal history of CN. The genetics of his CN were undefined until a repeat analysis was performed at 15 years of age and showed a pathogenic *SRP54* mutation. He began treatment with G‐CSF at 2 months of age and responded well (Figure 1A). He was largely managed with a dose of 3 μg/kg/day. He did not have any severe or life‐threatening infections and never required intravenous antibiotic therapy but did have recurrent mild infections treated with oral antibiotics (e.g., acute otitis media). He did not have malabsorption or growth failure and his weight ranged from the 34th to 91st percentiles throughout childhood. No dysmorphic features were present. He did require an individualized education program (IEP) at school for a mild neurodevelopmental delay, but no objective measures of cognitive delay were available.

Immunophenotyping of the patient's myeloblasts is described in Figure 1. Fluorescence in situ hybridization (FISH) identified a deletion of 5q31.2 *(EGR1)* in 68% of cells and an AML‐specific FISH panel for *RUNX1*, *KMT2A*, and *CBFB* was negative. Next generation sequencing performed by FoundationOne HEME identified mutations in *CSF3R* (Q776) and *RUNX1* (p113L). No other abnormalities were identified on cytogenetics or FoundationOne sequencing. The patient was diagnosed with acute myeloid leukemia (AML) with myelodysplasia‐related changes based on the 2016 World Health Organization myeloid neoplasm classification [6]. His G‐CSF was stopped and he began therapy with CPX‐351 (liposomal daunorubicin‐cytarabine). Deletion of 5q is a poor prognostic finding in AML and this patient's therapy was chosen based on superior outcomes using CPX‐351 compared to conventional chemotherapy in patients with AML and myelodysplastic syndrome (MDS)‐like changes [7]. He received one cycle of CPX‐351 as per the Children's Oncology Group study, AAML1831 consisting of 60 mg/m^2^ doses on day 1, day 3, and day 5. Overall, he tolerated this regimen well but developed significant gingivitis and periodontitis complicated by a hematoma. He received 7 days of intravenous vancomycin and tranexamic acid‐soaked gauze and these complications completely resolved upon count recovery. A repeat bone marrow analysis was performed 35 days after the start of CPX‐351 and showed a hypocellular marrow for age (45%–55% cellularity) but no morphologic evidence of leukemia. FISH did not identify the previously seen 5q31.2 deletion and minimal residual disease analysis by Hematologics (Seattle, WA, USA) was also negative for leukemia. The ANC at the time of these studies was 0.81 × 10^3^ cells/μl. He proceeded to HCT after cycle 1 of CPX‐351.

The patient received a peripheral blood stem cell (PBSC) graft from a 10/10 matched unrelated male donor after conditioning with busulfan (3.2 mg/kg/dose, four doses) and cyclophosphamide (50 mg/kg/dose, four doses). Graft versus host disease (GvHD) prophylaxis included cyclosporine, mycophenolate mofetil, and abatacept. Platelet and neutrophil engraftment occurred on day +12. Engraftment studies on day +15 showed >98% donor chimerism. Bone marrow studies on day +100 also showed full donor chimerism and no evidence of leukemia or myelodysplasia. He developed steroid‐responsive late onset acute GvHD of the skin and liver and chronic oral GvHD 7 months after HCT, but otherwise has not had any significant complications at a follow‐up time of 9 months. Table 1 compares this patient's HCT details to other published HCTs in patients with *SRP54* mutations.

**TABLE 1:** Demographics and hematopoietic cell transplant (HCT) details for our patient (patient 1) and other published reports of HCT in patients with SRP54 mutations

	Patient 1	Tamura et al. [3]	Carapito et al. [5]	Bellanne ´‐Chantelot et al. [1]	Carden et al. [4]
*SRP54* mutation(s)	p.Thr117del	p.Gly225Asp p.Gly274Asp	Patient 1: pGly226Glu Patient 2: pThr115Ala	Patient 1: p.Thr117del Patient 2: p.Gly226Glu	p.Thr117del
Other diagnoses	AML with myelodysplastic changes	None	Patient 1: none Patient 2: none	Patient 1: none Patient 2: none	Chromosome 22q11.2 deletion syndrome
Age at HCT, sex	15 years, male	8 months, female	Patient 1: 4 years, male Patient 2: 1 year, female	Patient 1: 1.5 years, male Patient 2: 0.5 years, male	2 years, male
Conditioning regimen	Busulfan, cyclophosphamide	Fludarabine, cyclophosphamide, etoposide, melphalan	Patient 1: not reported	Patient 1: not reported Patient 2: not reported	Alemtuzumab, fludarabine, melphalan
Donor, match	Unrelated, 10/10	Unrelated, mismatched	Patient 1: unrelated, genoidentical Patient 2: unrelated	Patient 1: not reported Patient 2: not reported	Sibling, 8/8
Graft	PBSC	Cord	Patient 1: not reported Patient 2: cord	Patient 1: not reported Patient 2: not reported	Cord
Cell dose Nucleated (cells/kg) CD34+ (cells/kg)	10.9 x 10^8^ 8.9 x 10^6^	2 x 10^8^ 6.3 x 10^5^	Patient 1: not reported Patient 2: not reported	Patient 1: not reported Patient 2: not reported	2 x 10^8^ 8.8 x 10^5^
GvHD prophylaxis	Cyclosporine, mycophenolate, abatacept	Tacrolimus, methotrexate	Patient 1: not reported Patient 2: not reported	Patient 1: not reported Patient 2: not reported	Cyclosporine, mycophenolate
Neutrophil engraftment	Day +12	Day +19	Patient 1: not reported Patient 2: not reported	Patient 1: not reported Patient 2: not reported	Day +14
Complications	Late acute GvHD Chronic GvHD	None	Patient 1: none Patient 2: VOD, death	Patient 1: not reported Patient 2: not reported	CMV viremia
Follow‐up time from HCT	9 months	14 months	Patient 1: 2 years Patient 2: death (2 months)	Patient 1: 9.5 years Patient 2: 1 year	6 months

Abbreviations: AML, acute myeloid leukemia; CMV, cytomegalovirus; GvHD, graft versus host disease; PBSC, peripheral blood stem cells; VOD, veno‐occlusive disease.

HCT is commonly used for the treatment of CNs. Indications include G‐CSF refractoriness or the development of MDS and/or acute leukemia. *SRP54*‐mutated CN was first described in 2017 [5] and a study of 23 patients with these mutations did not have single case of leukemia or MDS (median follow‐up time of 15 years) [1]. The in‐frame deletion seen in our patient (p.Thr117del) was the most common mutation in that study [1]. Deep sequencing of 17 patients in that cohort did not identify *CSF3R*, *RUNX1*, or *TP53* mutations, which have been implicated in leukemogenesis in CNs. Based on these observations, Oyarbide and Corey [8] questioned whether longer follow up would unveil a risk for leukemic transformation, potentially with different cooperating mutations. Our patient developed AML at 15 years of age and tumor sequencing identified *CSF3R* and *RUNX1* mutations. *CSF3R* mutations occur in up to 80% of CN patients who develop myeloid malignancies and commonly coexist with *RUNX1* mutations [9]. Skokowa et al. [9] studied 31 patients with CN and MDS/leukemia and found that 80.5% of patients with *RUNX1* mutations had concurrent *CSF3R* mutations, suggesting that these mutations together contribute to leukemic transformation in CNs. The presence of these leukemogenic mutations in our patient suggests overlap exists between the pathways of malignant transformation in *SRP54*‐mutated CN and more common causes of CN (e.g., *ELANE* and *HAX1* mutations). This is an important observation since *SRP54* mutations have not previously been linked to an increased risk of leukemia.

Although conclusions from this single report of AML in *SRP54*‐mutated CN must be limited, this finding has significant implications for the management of patients with *SRP54*‐mutated CN, particularly regarding screening practices and the role of HCT. A long‐term follow‐up study of patients with *SRP54* mutations is needed to further characterize the incidence and biology of leukemia in this patient population.

## AUTHOR CONTRIBUTIONS

Anthony Sabulski wrote the manuscript, collected data, and designed the table. David D. Grier provided pathology expertise and designed the figure. Kasiani C. Myers edited the manuscript and provided bone marrow failure syndrome expertise. Stella M. Davies edited the manuscript, collected data, and provided bone marrow failure syndrome expertise. Jeremy D. Rubinstein designed the study, provided leukemia expertise, and edited the manuscript.

## CONFLICT OF INTEREST

The authors have no conflicts or financial interests to disclose.

## ETHICS STATEMENT

This study describes the clinical treatment and outcomes of a patient who received care at our center. All ethical standards were complied with during this study. There is no identifying data included in the manuscript. No animal work was done in this study.

## FUNDING INFORMATION

No funding was received for this work.

## PATIENT CONSENT STATEMENT

The patient consented to the HCT tissue repository at our institution.
